# The dosimetric impact of control point spacing for sliding gap MLC fields

**DOI:** 10.1120/jacmp.v17i6.6345

**Published:** 2016-11-08

**Authors:** Benjamin J. Zwan, Jonathan Hindmarsh, Erin Seymour, Kankean Kandasamy, Kirbie Sloan, Rajesakar David, Christopher Lee

**Affiliations:** ^1^ Central Coast Cancer Centre Gosford Hospital Gosford NSW Australia; ^2^ School of Mathematics and Physical Sciences University of Newcastle Newcastle NSW Australia

**Keywords:** MLC, DLG, commissioning, IMRT

## Abstract

Dynamic sliding gap multileaf collimator (MLC) fields are used to model MLC properties within the treatment planning system (TPS) for dynamic treatments. One of the key MLC properties in the Eclipse TPS is the dosimetric leaf gap (DLG) and precise determination of this parameter is paramount to ensuring accurate dose delivery. In this investigation, we report on how the spacing between control points (CPs) for sliding gap fields impacts the dose delivery, MLC positioning accuracy, and measurement of the DLG. The central axis dose was measured for sliding gap MLC fields with gap widths ranging from 2 to 40 mm. It was found that for deliveries containing two CPs, the central axis dose was underestimated by the TPS for all gap widths, with the maximum difference being 8% for a 2 mm gap field. For the same sliding gap fields containing 50 CPs, the measured dose was always within ±2% of the TPS dose. By directly measuring the MLC trajectories we show that this dose difference is due to a systematic MLC gap error for fields containing two CPs, and that the cause of this error is due to the leaf position offset table which is incorrectly applied when the spacing between CPs is too large. This MLC gap error resulted in an increase in the measured DLG of 0.5 mm for both 6 MV and 10 MV, when using fields with 2 CPs compared to 50 CPs. Furthermore, this change in DLG was shown to decrease the mean TPS‐calculated dose to the target volume by 2.6% for a clinical IMRT test plan. This work has shown that systematic MLC positioning errors occur for sliding gap MLC fields containing two CPs and that using these fields to model critical TPS parameters, such as the DLG, may result in clinically significant systematic dose calculation errors during subsequent dynamic MLC treatments.

PACS number(s): 87.56.nk

## I. INTRODUCTION

In external beam radiotherapy, the multileaf collimator (MLC) is used to shape the X‐ray beam to produce irregular beam geometries.^1^, ^2^, ^3^ In advanced delivery techniques, such as intensity‐modulated radiotherapy (IMRT) and volumetric‐modulated arc therapy (VMAT), the MLC is employed to dynamically shape the beam during the delivery^4^, ^5^, ^6^, ^7^, ^8^ in order to achieve highly conformal dose distributions. Dynamic MLC (DMLC) techniques are available on all commercially available linear accelerators and have been widely adopted for the treatment of most disease sites.^9^, ^10^, ^11^

Accurate modeling of the MLC properties within the treatment planning system (TPS) is essential to ensure accurate dose delivery for IMRT and VMAT.^12^, ^13^, ^14^ For the Varian Eclipse TPS (Varian Medical Systems, Palo Alto, CA), the only MLC properties that may be edited by the user are the MLC transmission factor and the dosimetric leaf gap (DLG).^13^, ^14^ The DLG is a parameter used in Eclipse to model the difference between the light field edge and the radiation field edge. This difference is caused by X‐ray transmission through the rounded end of the MLC.^1^, ^7^, ^15^ Eclipse corrects for this by reducing the distance between opposing MLC leaves by a fixed amount at each control point in the plan. The change in MLC gap width is referred to as the DLG or the radiation field offset. Vial et al.^(13)^ investigated the validity of several experimental techniques to determine the DLG and concluded that sliding gap MLC fields^(16)^ should be used for measurement of the DLG.

Incorrect measurement of the DLG results in systematic MLC gap errors, where the distance between opposing MLC leaves at delivery differs from the distance calculated in the TPS. A number of groups have categorized the impact of these types of MLC errors in IMRT and VMAT for a range of treatment sites.^(4,17–21)^ Rangel et al.^(18)^ found that an MLC gap error of 1 mm resulted in a systematic dose difference of 2.7% and 5.6% for prostate and head and neck IMRT respectively. Other groups have reported that a systematic MLC gap error of 1 mm can introduce dose errors of up to 10% in IMRT treatments.^(21)^ Similarly, Oliver et al.^(20)^ reported an increase of 2.8 Gy per mm of MLC gap error for VMAT deliveries. It is clear from these works that any errors in measurement of the DLG can potentially result in clinically significant dose delivery errors.

The leaf position offset correction (LPO) is a correction which is applied by Varian treatment units to correct for the difference between the light field position and the MLC leaf tip position.^(13)^ This is required because the control points of the treatment plan specify the MLC‐defined light‐field position, whilst the MLC positions themselves are calibrated using the leaf tip positions. This correction is effectively a shift applied to the planned MLC positions which is zero on the central axis and increases with off‐axis distance (in the direction MLC motion).

In this investigation, we report on the detection of systematic MLC gap errors during the delivery of sliding gap MLC fields created using just two control points (CPs). We then demonstrate that these errors do not occur for identical fields with a smaller spacing of CPs and show that the cause of such errors is due to incorrect application of the LPO correction by the MLC control system. Following this, recommendations are made on the maximum spacing of CPs required to ensure accurate leaf positioning. We quantify how the detected errors can impact the measurement of the DLG, and show that this can translate into clinically significant dose delivery errors for IMRT and VMAT treatments.

## II. MATERIALS AND METHODS

### A. Sliding window output factor measurements

All measurements in this investigation were performed on a Varian 21iX linear accelerator equipped with a Millennium 120 leaf MLC (Varian Medical Systems, Palo Alto, CA). Sliding window MLC fields were created such that a fixed gap width was scanned across a symmetric $10\times 2\;{\text{cm}}^{2}$ jaw‐defined field. All MLC files were created in the Shaper computer program (Version 7.0, Varian Medical Systems) and were subsequently imported into an Eclipse treatment planning system (TPS) (Version 11.030, Varian Medical Systems). The predicted dose in a flat water phantom was calculated using the analytical anisotropic algorithm (AAA) for each field.

Sliding gap files were generated with gap widths of 2, 5, 10, 20 and 40 mm. Each field was delivered with three leaf speeds (5, 10 and 25 mm/s), two photon beam energies (6 MV and 10 MV), and at two depths (5 cm and 10 cm) in a plastic water phantom (Computerized Imaging Reference Systems, Norfolk, VA). All measurements were performed at 0° collimator and gantry rotation. For each delivery, the measured sliding window output factor (SWOF) was computed by taking a ratio of the output for the dynamic sliding gap field to the output for a reference $10\times 2\;{\text{cm}}^{2}$ field under the same setup conditions. Measurements were performed using a PTW TN30013 0.6cc Farmer type ionization chamber positioned at the machine isocenter and orientated with its long axis perpendicular to the direction of MLC motion. The TPS‐predicted SWOF was calculated for each delivered field using the same normalization technique, taking into account the effective point of measurement of the ionization chamber.

Two sets of SWOF measurements were performed. The first set consisted of the sliding gap fields listed above and contained CPs (i.e., with a CP at the beginning and end of the delivery). For the second set of measurements, the same sliding gap fields were created instead containing 50 evenly spaced CPs. These 50 CP fields were produced from the original two CP MLC Shaper files by linearly interpolating the leaf positions as a function of dose fraction using an in‐house software tool developed in the MATLAB programming language (MathWorks, Natick, MA). The measured SWOFs for each of these two sets were compared to the SWOFs predicted by the TPS. Note that the number of CPs (i.e., 50) was chosen arbitrarily to ensure a sufficiently small spacing between CPs. The DLG values used by the TPS to calculate the predicted SWOFs was 1.47 mm and 1.61 mm for 6 MV and 10 MV, respectively, which were calculated using MLC files with 50 CPs (see Materials and Methods section E for further details).

### B. Dose profile comparison

Central axis dose profiles were measured in the X direction (i.e., the direction of MLC travel) for the sliding gap fields described in Section A above for both 2 and 50 CP fields. The same monitor units were delivered for each field. These measurements were performed using a MapCHECK2 two‐dimensional diode array (Sun Nuclear Corporation, Melbourne, Fl) at 5 cm and 10 cm depth in a plastic water phantom with the plane of the detectors positioned at the machine isocenter. The sensitivity of each individual diode and any directional dependence was corrected using the array calibration function within the SNC Patient acquisition software (Sun Nuclear Corporation). Each set of diode measurements were converted to dose using central axis point dose measurements performed with an ionization chamber. The measured dose profiles were compared to the corresponding dose profiles extracted from the TPS for each sliding gap field.

### C. Measurement of MLC trajectories

MLC positions were measured as a function of time for each in‐field MLC during 1 cm sliding gap deliveries with 2, 3, 4, 5, 10 and 50 CPs. The purpose of this set of measurements was to systematically increase the density of the CPs in order to determine a minimum requirement for number of CPs during sliding gap deliveries.

MLC positions were measured using a Varian aS1000 electronic portal imaging device (EPID) operating in continuous acquisition (cine) mode. Individual EPID image frames were acquired at 8.5 frames‐per‐second using an external PC equipped with a frame grabber. The position of each MLC leaf was extracted from each frame by means of the methodology described by Fuangrod et al.,^22^, ^23^, ^24^ which was further developed by Zwan et al.^(25)^ For each image frame acquired, the horizontal beam profile through the central axis of each leaf pair was first extracted. For each in‐field MLC the location of the 50% radiation field edge was found to subpixel accuracy using cubic‐spline interpolation. Additional details and validation of this methodology can be found in Zwan et al.^(25)^ The MLC gap was found by calculating the distance between each opposing leaf pair. The off‐axis distance was taken to be the distance from the average position of the two MLCs (i.e., the center of the gap) to the central pixel of the imaging panel. The measured MLC gap error as a function of off‐axis distance was computed using the difference between the average MLC gap from each image frame compared to the average MLC gap for a static MLC field on the central axis. Image analysis was performed automatically using MATLAB software to extract the MLC positions. Note that, using the EPID to determine the MLC positions is a measurement of the true radiation field edge and is independent of the original treatment plan and the MLC control system. For this reason EPID‐based MLC position measurements were used as a gold standard in this investigation.

### D. Simulation of leaf position offset errors

Errors were intentionally introduced into the LPO table in order to demonstrate that the detected MLC gap errors were a result of an incorrect interpolation of the LPO correction in between control points. The LPO correction table is stored within the MLCTABLE.txt file located on the 4DITC computer of the linear accelerator control system. This file contains the LPO as a function of off‐axis distance and is loaded into the MLC controller during the initialization process.

Errors were introduced into the table such that the LPO correction at all off‐axis positions was the same and equal to the LPO at the location of the control points of the two CP sliding gap fields. A 1 cm sliding gap field containing 50 evenly spaced control points was then delivered before and after altering the table and, in each case, the MLC gap was measured as function of time using cine EPID imaging. During these deliveries, the machine log files (DynaLog files; Varian Medical Systems) were also recorded and also used to assess the MLC gap versus off‐axis distance.

### E. Measurement of the dosimetric leaf gap

The DLG can be determined using dynamic sliding gap fields as described by Vial et al.^(13)^ In this method, the DLG is measured on the central axis using a series of sliding gap fields of different gap widths (6 mm up to 20 mm) each delivered with the monitor units required to achieve the same dose as an open $10\times 2\;{\text{cm}}^{2}$ static field. Each MLC file is edited to contain MLC position offsets of 0.0,‐0.4,‐0.8,‐1.2, and ‐1.6 mm at each CP. The output measured for each field can be used to find the optimal MLC position offset which minimizes the dose difference for all gap widths. Note that a range of gap widths are used here as the DLG is dependent of the distance between opposing MLC leaves. This optimal offset is indicative of the measured DLG. In this work, the DLG was determined using this method for files containing 2 CPs ${(\text{DLG}}_{2\text{CP}})$ and 50 CPs ${(\text{DLG}}_{50\text{CP}})$ separately.

### F. Impact on IMRT dose calculations

The 3D dose distribution was calculated in the TPS for a clinical 10 MV Pelvis IMRT treatment plan using ${(\text{DLG}}_{2\text{CP}})$ and ${(\text{DLG}}_{50\text{CP}})$ with a 1 mm grid size. The two dose distributions were then compared volumetrically by means of a dose‐volume histogram (DVH) analysis of the planning target volume (PTV) and critical organs at risk (OARs).

## III. RESULTS

### A. Measured dose differences

**Figure 1:**
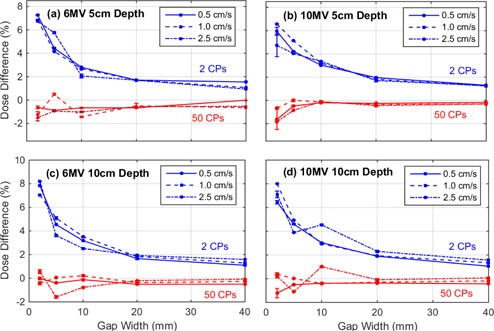
The percentage difference between measured point dose and TPS point dose on the central axis as a function of nominal MLC gap width for (a) 6 MV at 5 cm depth, (b) 10 MV at 5 cm depth, (c) 6 MV at 10 cm depth, and (d) 10 MV at 10 cm depth in a flat water phantom. The difference is displayed for fields delivered with three different leaf speeds and for MLC files containing both 2 and 50 CPs. All data points in this plot represent the mean of two repeated readings. The amplitude of the range bars is equivalent to the difference in dose errors calculated from either reading.

SWOFs were measured on the central axis and compared to the TPS for fields containing both 2 CPs and 50 CPs. Figure 1 displays the difference between the measured and TPS‐predicted SWOFs as a function of nominal MLC gap width for 6 MV and 10 MV photon beams at 5 cm and 10 cm depth in a plastic water phantom. The measurements were repeated twice and the mean of the two measurements are represented by the data point in Fig. 1. The range between the two measured SWOF errors are represented by the uncertainty bars associated with each data point.

### B. Dose profile comparison

**Figure 2:**
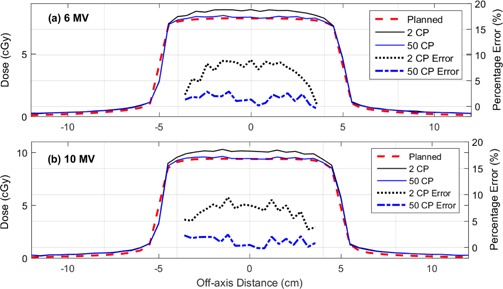
Comparison between measured and TPS‐predicted x‐axis profiles for a 5 mm sliding gap delivery for fields containing both 2 and 50 CPs. Data are displayed for (a) 6 MV and (b) 10 MV beams at 10 cm depth in a plastic water phantom. The in‐field percentage dose difference between the measured and TPS‐planned profile has also been plotted for the 2 and 50 CP deliveries.

In order to further investigate the discrepancy in dose delivered by the 2 and 50 CPs fields, dose profiles were measured using a diode array in a plastic water phantom and compared to the corresponding TPS dose profile. Figure 2 shows the 2 and 50 CP x‐axis profile for a 5 mm sliding gap delivery at 10 cm depth for (a) 6 MV and (b) 10 MV. In each case, the two measured profiles are compared to the corresponding TPS‐predicted beam profile.

### C. Measured MLC gap errors

**Figure 3:**
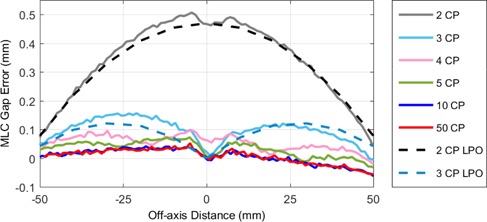
The MLC gap error as a function of MLC position in the direction of travel for a 6 MV sliding gap delivery with a nominal gap width of 1 cm and a leaf speed of 1 cm s^‐1^. The gap error is plotted for fields containing 2, 3, 4, 5, 10, and 50 CPs. The dashed lines represent the predicted leaf gap error calculated using the LPO table.

Using the MLC positions extracted from cine EPID images, the gap width between opposing leaf pairs was calculated and compared to the expected gap width. The expected gap width was determined using an integrated EPID image of a static MLC‐defined field on central axis. Figure 3 shows a plot of the expected and planned gap width (i.e., the gap with error) as a function of off‐axis distance for a 1 cm sliding gap delivery with 2, 3, 4, 5, 10, and 50 CPs. Each data point represents an average over all in‐field leaf pairs.

### D. Simulation of leaf position offset errors

**Figure 4:**
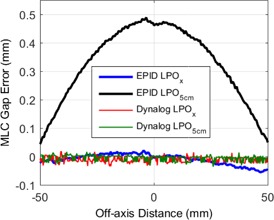
The MLC gap error as a function of MLC position in the direction of travel for a 6 MV sliding gap delivery with a nominal gap width of 1 cm and a leaf speed of 1 cm s^‐1^. Gap errors were measured during two subsequent deliveries. For the first delivery, the standard LPO correction table was applied (${\text{LPO}}_{x}$). For the second delivery, LPO correction table was edited (${\text{LPO}}_{5\text{CM}}$) so that the LPO at all off‐axis distances was equal to the LPO at the control points (i.e., at 5 cm off‐axis). The MLC gap errors were measured using EPID images and extracted from DynaLog files.

Figure 4 shows the measured MLC gap errors for two deliveries of the 1 cm sliding gap field with 50 CPs. During the first delivery the LPO table was unaltered, which is referred to as ${\text{LPO}}_{x}$ in Fig. 4. During the second delivery, the LPO was edited such that the LPO correction value at all off‐axis positions was equal to the LPO at 5 cm off‐axis. This delivery is referred to as ${\text{LPO}}_{5\text{CM}}$ in Fig. 4. Note that both deliveries contained 50 CPs, and the only variable that was changed between the two measurements was the LPO correction table. Figure 4 contains gap errors which were calculated using MLC positions from both EPID and DynaLog file measurements. For the DynaLog‐based measurements, the gap error was computed using the difference between the measured and planned MLC positions recorded in the DynaLog files.

### E. Measurement of the dosimetric leaf gap

The DLG was measured using sliding gap fields for MLC files containing 2 CPs (${\text{LPO}}_{5\text{CM}}$) and 50 CPs (${\text{LPO}}_{50\text{CM}}$) for 6 MV and 10 MV beams. The DLG values obtained by each set of measurements are given in Table 1.

**Table 1:** Measured values for ${\text{DLG}}_{2\text{CP}}$ and ${\text{DLG}}_{50\text{CP}}$ obtained using the DMLC method for 6 MV and 10 MV photon beams

*Energy*	${\text{DLG}}_{2\text{CP}}$ *(mm)*	${\text{DLG}}_{50\text{CP}}$ *(mm)*	*Diff. (mm)*
6 MV	1.98	1.47	0.5 (34%)
10 MV	2.11	1.61	0.5 (31%)

### F. Dosimetric errors in IMRT deliveries

**Figure 5:**
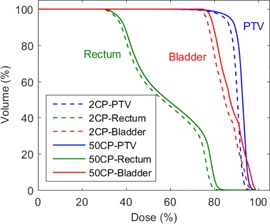
DVH plot for the TPS‐calculated dose for a 10 MV clinical IMRT pelvis treatment plan showing the dose to the PTV, bladder, and rectum. The solid lines indicate the calculated dose using ${\text{DLG}}_{50\text{CP}}$ and the dashed lines indicate the calculated dose using ${\text{DLG}}_{2\text{CP}}$.

Figure 5 shows a DVH of the PTV and critical OARs for a 10 MV clinical IMRT treatment plan. The DVH is plotted for the TPS‐predicted dose using ${\text{LPO}}_{2\text{CM}}$ (2.11 mm) and ${\text{LPO}}_{50\text{CM}}$ (1.61 mm). The mean PTV dose difference between these two calculations was ‐1.4Gy (‐2.6%) over the treatment course. The purpose of these two calculations was to quantify how much the measured change in DLG would impact a complex IMRT dose calculation in our TPS.

## IV. DISCUSSION

For sliding gap fields containing two CPs, the TPS underpredicts the SWOF for all delivered fields (see Fig. 1). The difference between measurement and TPS increases with decreasing gap and was not found to be dependent on beam energy, depth of measurement, or MLC leaf speed. SWOFs measured for sliding gap fields containing 50 CPs gave better agreement with the TPS for all gap widths and were within ±2% for all fields. The range bars associated with the measured data points in Fig. 1 indicate that this conclusion is statistically significant over all the SWOFs measured. The uncertainty for most of the measured values was within 0.2% of the mean values and the maximum range was 1.95%, which was for the smallest gap fields (i.e., 2 mm). The discrepancy between measured and predicted dose is also evident in Fig. 2, which shows that the spacing between CPs impacts the dose delivered within the entire high‐dose region of the profile. The TPS‐calculated point dose and beam profiles were found to be identical for both 2 and 50 CPs, which is why only one TPS profile is displayed in each plot of Fig. 2.

Direct measurement of the MLC positions indicated that there was a 0.5 mm systematic MLC gap error on the central axis for fields containing two CPs. Figure 3 illustrates this and also shows that the magnitude of the error decreases as the MLCs move off axis. This gap error was not present for fields containing 50 CPs where the distance between opposing MLCs was always within ±0.06mm of the expected gap (see Fig. 3).

We provide evidence that this error is a result of the LPO correction, which is incorrectly applied when only two CPs are used. The LPO correction is a shift applied to the nominal MLC positions to account for the difference between the light field position and the MLC leaf tip position. The LPO is zero on the central axis and increases with off‐axis distance. For the two CP sliding gap fields in this work, the CPs are at ±5cm where the LPO correction is approximately 0.25 mm. If the LPO correction is applied to the MLC positions at the CPs prior to interpolation by the MLC control system, then all MLC positions (regardless of off‐axis position) would be retracted by the LPO at 5 cm (i.e., by 0.25 mm). On the central axis the MLC gap would then be increased by 2×0.25mm when ideally it should be unaffected by the LPO. This produces a gap error on central axis, which decreases in magnitude as the MLCs approach the CPs, where the correct LPO is applied. This off‐axis dependency of the gap error is seen in Fig. 2, which includes the percentage errors between the measured and planned dose over a range of off‐axis distances in the direction of leaf travel for both two CP and 50 CP fields. It can be seen that the error for deliveries with two CPs is highest on the central axis and decreases at the edges of the field (i.e., close to the CPs). The percentage errors for fields delivered with 50 CPs do not replicate this pattern and any dose errors do not appear to vary as a function of off‐axis distance.

The resultant gap error for the two CP fields, ${\text{Er}}_{\text{gap}}$, can therefore be predicted by the LPO table as a function of off‐axis distance, x, using Eq. (1) below.

(1) $$Er_{gap}(x)=2\times [LPO(5cm)-LPO(x)]$$

This predicted gap error is plotted in Fig. 3 for both two CP and three CP and qualitatively agrees with the measured error. Note that, the equation above only applies to fields with two CPs; however, a similar equation could be derived for any given number of control points. Equation (2) details a general formula for the gap error, as a function of off‐axis distance, x, between any two control points at distances, $x_{1}$and $x_{2}$from the central axis. This equation reduces to Eq. (1) when $x_{1}=+5\text{cm}$ and $x_{2}=‐5\text{cm}$.

(2) $$Er_{gap}(x)=2\times \left[\left\{\frac{LPO(x_{2})-LPO(x_{1})}{x_{2}-x_{1}}\right\}\times (x-x_{1})+LPO(x_{1})-LPO(x)\right]$$

The predicted errors were not plotted for 4, 5, and 10 CPs as the relative magnitude of these errors is extremely small (<50μm).

It is worth noting that the predicted gap errors adhere to the same trends as the measured errors. Firstly, the number of peaks in each curve is equal to the number of CPs minus 1 which is due to the fact that the error is minimized in the areas surrounding the CPs. Secondly the magnitude of the error decreases as the spacing between CPs decreases. The reason for this is that the linear interpolation of the LPO values between CPs becomes a better approximation to the parabolic LPO curve when the CPs are closer together.

It can be seen that for all fields with a control spacing greater than 1 cm there is an observable MLC gap error. Based on this, the authors recommend a maximum CP spacing of 1 cm be used for sliding gap fields to ensure a consistent gap width at all off‐axis distances. It is important to note that this error does not occur for dynamic fields created within the TPS (e.g., sliding window IMRT) as the TPS automatically uses a sufficiently small CP spacing.

Figure 4 shows measured gap errors for a normal 50 CP delivery ${(\text{LPO}}_{x})$ and a 50 CP delivery with errors in the LPO table (${\text{LPO}}_{5\text{cm}}$). The ${\text{LPO}}_{5\text{cm}}$ delivery simulates the LPO errors that would occur for a two CP delivery, where the LPO at 5 cm is applied throughout the entire delivery. The EPID‐measured MLC gap errors in Fig. 4 show that the errors for two CP deliveries can be reproduced for deliveries with a high CP spacing simply by altering the LPO correction table. This is further evidence that incorrect interpolation of the LPO table is the cause of the observed SWOF errors and MLC gap errors.

Figure 4 also shows that, for the ${\text{LPO}}_{5\text{cm}}$ delivery, no MLC positioning errors were detected using DynaLog files, despite the fact that known positioning errors were introduced into the LPO table and validated using EPID measurements. The reason for this is that both the planned and measured MLC positions recorded within the DynaLog files are sourced from the MLC controller itself. Rather than being a true measurement of the MLC leaf position, the DynaLog files contain signals from the encoder of each MLC motor which has been converted to position. If an error is introduced into the planned MLC positions prior to communication with the MLC controller, then this will not be detected by comparing the planned and measured positions within the DynaLog file. Examples of such errors may include: manual editing of MLC calibration files on the 4DITC computer (as demonstrated in Fig. 4), MLC calibration errors,^(26)^ and general communication issues between the linac control system and the MLC controller. For the reasons discussed above, the authors recommend the use of more independent tools which rely on actual MLC‐defined radiation or light‐field edges for validation of dynamic MLC positions (e.g., cine EPID imaging). Furthermore, if DynaLog files are used then perhaps a more independent methodology would be to compare the measured MLC positions to that of DICOM plan file (directly exported from the TPS), rather than comparing to the planned positions within the DynaLog file itself. The error detected in this work is an example of a potential MLC error which would not have been detected using DynaLog file analysis alone.

The MLC gap errors result in an increase of 0.5 mm in the measured DLG when using fields with two CPs at ± 5 cm. The propagation of this error into the DLG measurement during IMRT commissioning would act to decrease in the gap between opposing MLC leaves during all subsequent dynamic MLC deliveries, resulting in a systematic decreases in the calculated dose of clinical plans.^(20)^ An example of one such error is demonstrated in Fig. 5 which shows that an error in the DLG, due to insufficient number of CPs, would result in an underestimation of dose by the TPS of ‐2.6% for the PTV. This demonstrates that using fields with only two CPs to determine the DLG can result in clinically significant errors during IMRT and VMAT commissioning. Note that, traditional pretreatment QA was performed for the ${\text{DLG}}_{50\text{CP}}$ treatment plan, which involved a 2D dose measurement and gamma comparison within a QA phantom using a 95% pass/fail criteria at 3%/3 mm (dose tolerance/distance‐to‐agreement). The details of this verification are not included here as the accuracy of IMRT dose delivery is not the focus of this work.

Some studies have suggested that one viable method for fine‐tuning of IMRT dose delivery is to adjust the MLC properties within the TPS (e.g., the DLG and MLC transmission factor) in order to achieve agreement between measured and planned IMRT dose distributions.^12^, ^27^, ^28^ It has also been shown that different combinations of these parameters can still result in acceptable agreement between the TPS and measurements for some fields.^(12)^ During IMRT commissioning this may lead to situations where an incorrect DLG is compensated for by adjusting other TPS parameters (e.g., the MLC transmission) to an incorrect value.^(13)^ If the DLG was measured using too large a spacing of CPs, then such a situation may arise in order to compensate for the error in the DLG. Subsequent adjustment of the TPS parameters to compensate for this error would result in dosimetric errors in some clinical situations (e.g., highly modulated IMRT fields with large amounts of MLC transmitted radiation). Previous authors have strongly recommended that the DLG should be measured in isolation rather than using IMRT treatment beams.^(13)^ In this work, we provide a methodology required to correctly measure the DLG which will reduce the need for time‐consuming and potentially erroneous optimization using IMRT treatment beam dose measurements.

Sliding gap fields are also used as a tool to assess the accuracy and constancy of the IMRT dose calculation within the TPS.^12^, ^29^ If an insufficient CP spacing was used in these deliveries, then the resultant MLC positioning error could potentially mask or compensate for real delivery errors or TPS errors (e.g., an MLC calibration error, DLG error, or MLC transmission factor error) if they induce a dose difference in the opposite direction. These errors have the potential to remain undetected during MLC commissioning and ongoing quality assurance, and may result in systematic delivery errors for all subsequent IMRT and VMAT treatments.

## V. CONCLUSIONS

The use of sliding gap MLC fields created using two CPs results in systematic MLC gap errors. These errors are at a maximum on the central axis and decrease as the MLCs move off‐axis, producing differences between the measured and TPS‐predicted dose of up to 8%. The cause of these errors has been shown to be an incorrect application of the LPO correction by the MLC control system. It has also been demonstrated that using sliding gap fields with two CPs will cause an over‐estimation of the measured DLG which may result in clinically significant dosimetric errors for dynamic MLC deliveries. The authors recommend that the maximum spacing between CPs should be 1 cm for sliding gap deliveries in order to ensure accurate MLC positioning and to avoid potentially significant errors during dynamic MLC commissioning.

## COPYRIGHT

This work is licensed under a Creative Commons Attribution 3.0 Unported License.

## Supporting information

Supplementary Material

Supplementary Material
