# Supplementary figures and images for: The dosimetric impact of control point spacing for sliding gap MLC fields
Source: J Appl Clin Med Phys. 2016 Nov 8;17(6):204–16. doi: 10.1120/jacmp.v17i6.6345 (PMC5690523; doi:10.1120/jacmp.v17i6.6345)

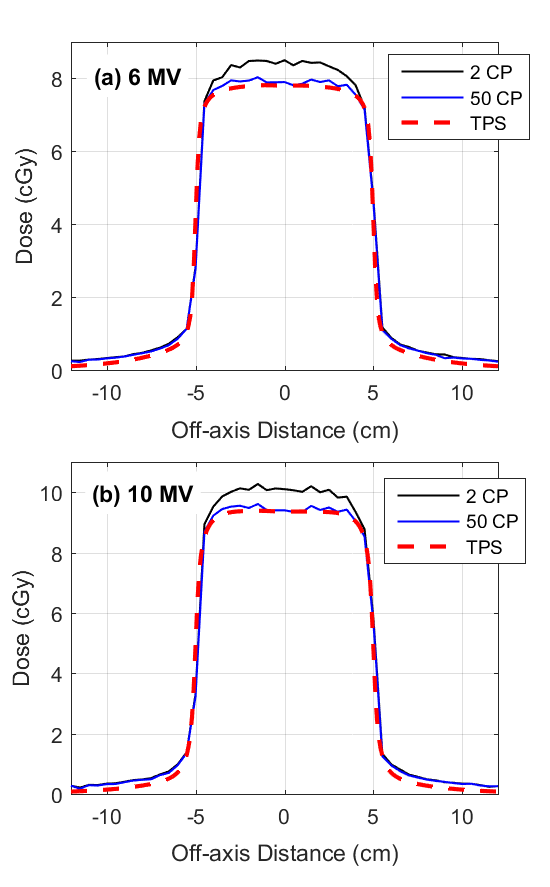

Supplement: Supplementary file 1 — Supplementary Material [file ACM2-17-204-s001.png]

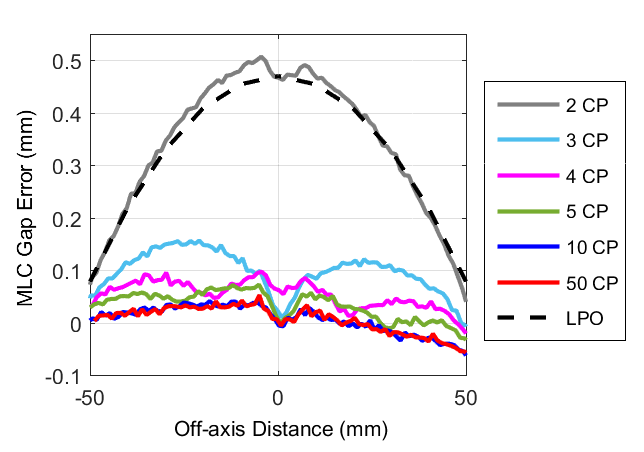

Supplement: Supplementary file 2 — Supplementary Material [file ACM2-17-204-s002.png]
